# Esthetic Management of Maxillary Midline Diastema Using Porcelain Laminate Veneers: A Case Report

**DOI:** 10.7759/cureus.91586

**Published:** 2025-09-04

**Authors:** Takoua Barhoumi, Zeineb Riahi, Linda Ayedi

**Affiliations:** 1 Department of Fixed Prosthodontics, Faculty of Dental Medicine of Monastir, Monastir, TUN

**Keywords:** central incisor, dental fluorosis management, dental veneers, diastema, esthetic dentistry

## Abstract

Maxillary midline diastema (MMD) is a common esthetic concern that can affect smile harmony and patient self-esteem, especially in cases involving intrinsic enamel defects like dental fluorosis. Managing diastema in fluorosed teeth presents clinical challenges due to enamel alteration and reduced bonding efficacy. This case report describes the esthetic rehabilitation of a 28-year-old male patient with a 4 mm diastema and moderate fluorosis-related discoloration of the maxillary anterior teeth. The patient had previously undergone unsuccessful orthodontic treatment. A minimally invasive treatment plan was developed involving porcelain laminate veneers (PLVs) placed on the four maxillary incisors. Diagnostic wax-up, mock-up, and digital impressions were used to guide the enamel-preserving tooth preparation. These steps also facilitated the precise fabrication of the veneers. The restorations were bonded using an adhesive protocol adapted to fluorosed enamel. The treatment resulted in successful diastema closure, harmonious tooth proportions, shade improvement, and high patient satisfaction. This case illustrates the value of PLVs as a conservative, effective, and esthetically reliable solution for midline diastema closure in a patient with moderate dental fluorosis and a history of failed orthodontic treatment, highlighting the importance of restorative alternatives when conventional approaches are unsuccessful.

## Introduction

Maxillary midline diastema (MMD), characterized as a space greater than 0.5 mm between the maxillary central incisors, is a common esthetic concern that can significantly impact an individual's self-esteem [1]. It can arise from various etiological factors, including discrepancies in tooth size and jaw dimensions [2,3], microdontia, mesiodens, an enlarged frenum or high frenal attachment [4-6], and abnormal oral habits [4]. Porcelain laminate veneers (PLVs) have proven to be a reliable and conservative solution for such cases, offering excellent esthetics, biocompatibility, and long-term stability, particularly for patients seeking minimally invasive treatment options [7,8]. Furthermore, advances in adhesive dentistry and ceramic technology have made it possible to achieve highly esthetic outcomes with minimal tooth reduction, preserving sound dental structure [2].

Although PLVs have shown high success rates for diastema closure, limited data exist on their effectiveness in complex cases, such as those involving enamel defects like fluorosis or a history of failed orthodontic treatment. This case is particularly relevant because it combines both conditions, making the treatment more challenging. In this context, PLVs were chosen over orthodontic retreatment or direct composite restorations because they provided a conservative solution capable of closing the diastema while simultaneously masking fluorosis-related discoloration.

This case report illustrates the esthetic and functional management of MMD. It highlights the diagnostic approach, preparation technique, and final outcome of treatment with PLVs. This case also adds a clinical example supporting their use in similar complex esthetic scenarios.

## Case presentation

A 28-year-old male patient presented to the Department of Fixed Prosthodontics at the Dental Clinic of Monastir with complaints of discolored teeth and spacing between his upper front teeth. He expressed a desire for a long-term esthetic correction, primarily focused on improving his smile. The patient’s dental history indicated a previous orthodontic treatment, but the diastema reappeared due to relapse despite retention, which was the main reason for its persistence. The patient was unwilling to undergo another prolonged orthodontic treatment because of time constraints and esthetic expectations.

The patient also reported a previous frenectomy performed before his orthodontic treatment, eliminating the need for further surgical intervention.

**Figure 1:**
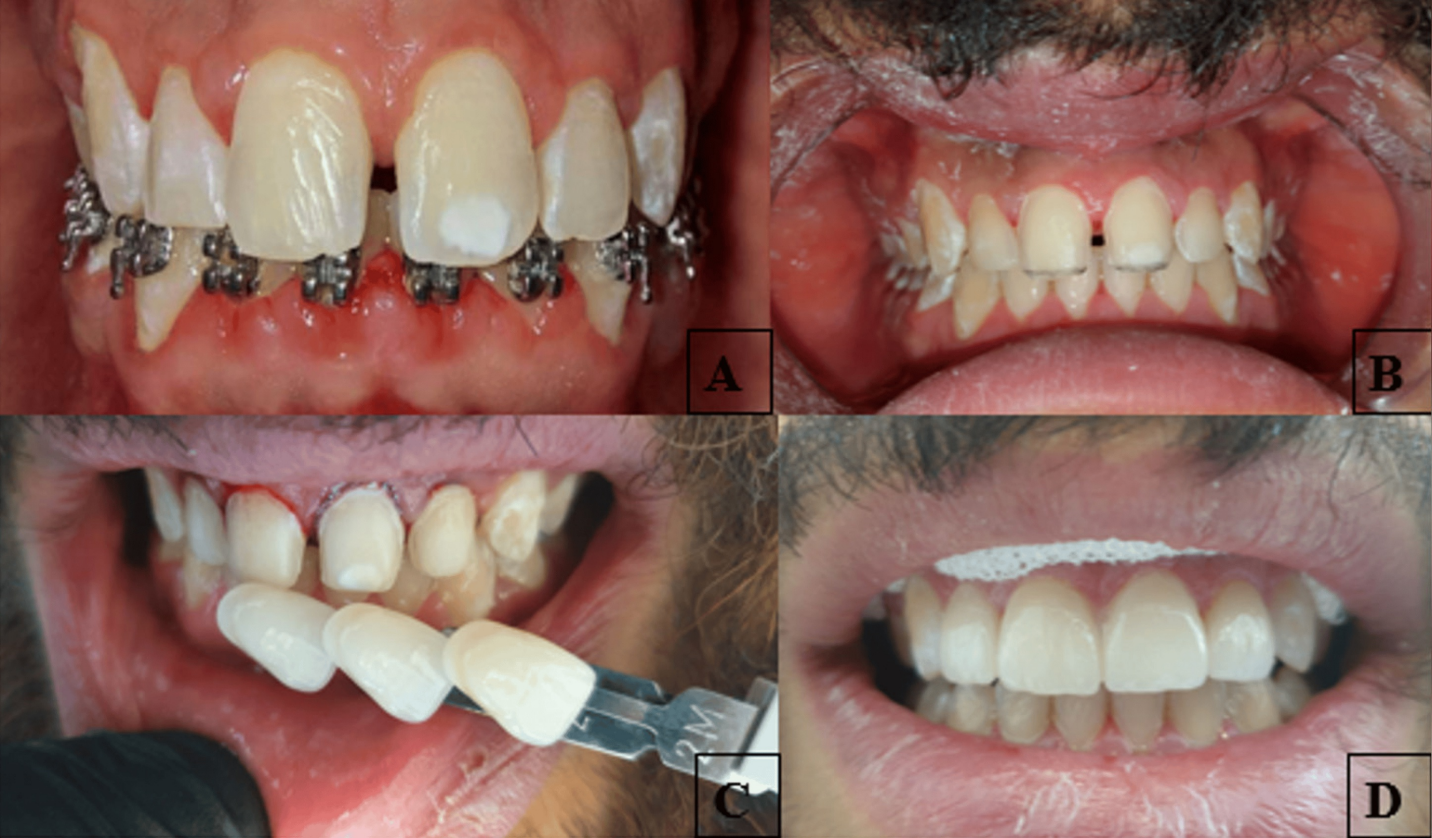
Clinical steps in the management of maxillary midline diastema using porcelain laminate veneers (A) Preoperative intraoral view showing a 4 mm maxillary midline diastema and fluorosis-related discoloration of the central incisors. (B) Tooth preparation limited to the enamel with a minimally invasive approach, preserving the tooth structure. (C) Shade selection using the VITA 3D-Master® ceramic shade guide (Vita Zahnfabrik, Germany) to ensure esthetic integration of the veneers. (D) Intraoral frontal view of the porcelain laminate veneers during the try-in phase, verifying fit, color match, and esthetic outcome.

Clinical examination identified a 4 mm diastema between the maxillary central incisors, a 3 mm overbite and overjet, and a Class I molar-canine relationship. The mandibular incisors were intact, showing no signs of wear, and no parafunctional habits were reported. Notably, the patient exhibited generalized enamel opacities and occasional white spots, consistent with very mild dental fluorosis, classified as score 2 on the Dean’s Index. The fluorosis was characterized by generalized opacities with scattered white spots predominantly on the labial surfaces of the anterior teeth (Figure 1A).

Baseline evaluation revealed healthy periodontal tissues, satisfactory oral hygiene, and no active carious lesions. No signs of temporomandibular joint (TMJ) or functional disturbances were noted.

After thorough clinical and radiographic evaluations, a minimally invasive treatment plan was developed involving the placement of PLVs on the four maxillary incisors. Written informed consent was obtained from the patient for both treatment and publication.

Study impressions were taken, and a diagnostic wax-up was performed to visualize the ideal tooth alignment and esthetic outcome. To further enhance esthetic planning and patient communication, Digital Smile Design (DSD) (São Paulo, Brazil) was utilized prior to the mock-up phase, helping to simulate the final outcome and align it with the patient’s esthetic expectations. The DSD analysis confirmed the absence of significant Bolton’s discrepancy and guided tooth proportion adjustments during the wax-up phase. Although strict golden proportion ratios were not fully achievable due to the patient’s natural tooth morphology, proportional harmony was ensured through DSD-guided modifications, resulting in a natural and esthetically pleasing outcome.

A preliminary mock-up, using bis-acrylic resin, allowed the patient to preview the final result and provide informed consent. The maxillary incisors were then conservatively prepared, maintaining enamel integrity with a depth of 0.5 mm using a depth-cutting diamond bur and tapered diamond bur (1 mm tip width) (Intensiv SA, Montagnola, Switzerland). The enamel preparation was limited to approximately 0.3-0.5 mm on the labial surface and extended interproximally beyond the contact area to allow for seamless veneer blending. A deep chamfer finish line was established at the gingival margin (Figure 1B).

Then, a gingival retraction was performed to improve the cervical contour and ensure a smooth esthetic transition between the veneer margins and surrounding soft tissues. A digital impression of the maxillary and mandibular arches was made with an intraoral scanner (TRIOS®; 3Shape, Copenhagen, Denmark). Using the shade guide of VITA 3D Master® (Vita Zahnfabrik, Germany), shade 2M2 was selected (Figure 1C).

PLVs were constructed, and the restorations were tried in for shade, fit, marginal adaptation, symmetry, size, contact, and shape. Initially, they were tried in individually, followed by a collective try-in to evaluate the overall esthetic integration. Patient approval was sought during the try-in process (Figure 1D).

To achieve bonding, we first proceeded with the veneers treatment: the veneers were arranged on a high viscosity silicone index, which indicated the precise tooth placement within the dental arch. This step was taken to prevent incorrect placement and accidental breakage. It was also essential to protect the labial surfaces of the veneers during the procedure. Direct exposure to acid can damage these surfaces, leading to their deterioration. 

**Figure 2:**
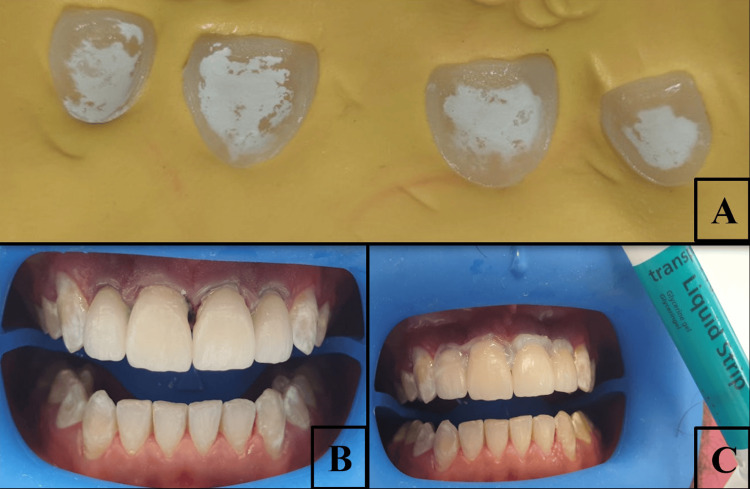
Bonding procedure of porcelain laminate veneers (A) Chalky white surface of the veneers observed after application of hydrofluoric acid, indicating successful surface etching. (B) Placement of veneers using adhesive luting resin, ensuring proper alignment and seating. (C) Application of glycerine gel on the margins prior to final light curing to prevent the formation of an oxygen-inhibited layer.

The inner surfaces of the PLVs were subjected to etching with hydrofluoric acid for 20 seconds. After etching, they were rinsed thoroughly using water and then dried (Figure 2A).

After that, a coat of silane was applied. The tooth treatment consisted of applying a 37% phosphoric acid for 30 seconds for enamel and 15 seconds for dentin. After rinsing and air drying, a thin layer of adhesive was applied. 

The composite cement was applied onto the internal surface of the veneers, and they were carefully positioned on the preparations (Figure 2B).

The laminate veneers were initially spot-cured for five seconds. Excess cement was removed with a probe, followed by final light curing for 20 seconds. During light curing, a glycerin gel was applied to the cervical margins to prevent the formation of an oxygen-inhibited layer between the composite material and the ceramic restorations (Figure 2C).

**Figure 3:**
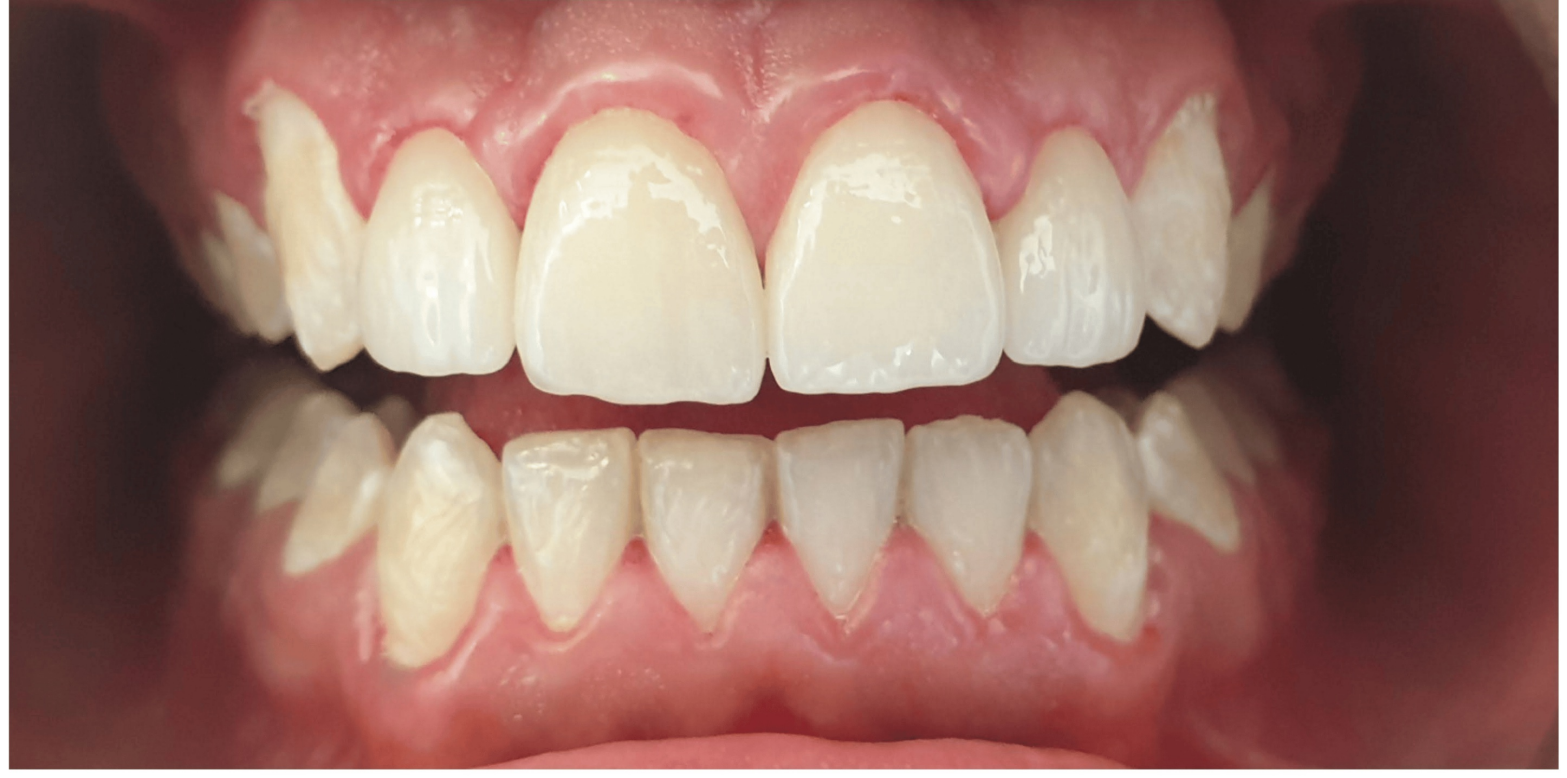
Final result after one week Postoperative intraoral view one week after cementation showing harmonious gingival integration, stable diastema closure, and natural esthetic outcome with well-adapted porcelain laminate veneers.

After the bonding procedure, diamond burs, polishing discs, and silicone polishers (Sof-Lex Finishing and Polishing System; 3M ESPE, St. Paul, USA) were used to finish the veneers. The patient was recalled for a follow-up appointment after one week to assess static and dynamic occlusion and to refine the polish of the restorations (Figure 3).

## Discussion

MMD is most frequently observed during the mixed dentition phase, but its persistence into adulthood often requires therapeutic intervention due to both esthetic and psychosocial implications [1].

The prevalence of MMD varies across populations and is influenced by factors such as age, ethnicity, and dental arch morphology [9]. A recent cross-sectional study in the Kurdistan region of Iraq found a notably high prevalence of MMD at 23.2%. The diastema was predominantly located in the maxilla (97%), significantly more prevalent in female individuals (26.4%) than male individuals (20.3%), and most common among ≥ 30 years old (55.8%) and children under 15 (37.7%) [10].

Because of the potential for multiple etiologies, the diagnosis process for MMD must be based on a thorough dental and medical history, clinical examination, occlusal analysis, and radiographic assessment [4].

Treatment planning for diastema correction, including orthodontic closure, restorative therapy, surgical correction, or a multidisciplinary approach, should consider both the underlying cause and the patient’s esthetic demands [2].

Orthodontic treatment is traditionally the first-line option, especially in growing patients with adequate crown proportions. However, orthodontic correction is often time-intensive, costly, and carries a significant risk of relapse without long-term retention [11]. As a result, restorative options have gained popularity for adult patients seeking faster and more predictable esthetic outcomes. The restorative closure of diastema can be achieved by using direct composite veneers, indirect composite veneers, PLVs, all-ceramic crowns, metal-ceramic crowns, and composite crowns [2,4].

A small diastema (1-1.5 mm) can be closed with direct composite resin restorations, using microfilled and hybrid resin [2]. It offers affordability and conservative management, is easy to use, and requires fewer appointments; however, it is highly technique-sensitive, offers less wear resistance and surface staining, which makes it inferior to dental porcelain. Clinical studies have reported that up to 38% of composite diastema restorations show discoloration within five years post-treatment, necessitating periodic maintenance or surface polishing [2,12].

Full-coverage crowns, although effective for masking discolorations or correcting shape and alignment issues, are considered overly invasive for diastema closure in teeth with otherwise healthy structure [4]. PLVs have become a widely accepted alternative to direct composite restorations, full-coverage ceramic crowns, and traditional porcelain-fused-to-metal (PFM) prostheses [2].

Given the patient’s history of failed orthodontic treatment, enamel discoloration due to fluorosis, and a 4 mm diastema, PLVs were selected for their ability to meet both functional and esthetic demands while allowing for conservative tooth preparation (emplacement).

Functionally, PLVs offer a remarkable balance between mechanical performance and esthetic result. Their durability and fracture resistance have been well-documented, with long-term clinical studies reporting survival rates exceeding 90% over a period of 10 years [4,13,14]. 

One of the major advantages of PLVs lies in their superior optical properties. Due to their translucency, fluorescence, and ability to reflect and transmit light similarly to natural teeth. They can mimic the appearance of natural teeth more closely than most other restorative materials, making them an ideal option for anterior restorations where esthetics is paramount [2,7,15].

In addition to esthetics, PLVs demonstrate excellent gingival biocompatibility. Their highly glazed ceramic surface reduces plaque accumulation and promotes healthy soft tissue response. Furthermore, their resistance to extrinsic staining ensures long-term color stability, a key limitation of resin-based composites [2].

Despite their advantages, PLVs have limitations. Therefore, careful case selection remains essential, considering factors such as sufficient enamel presence, extent of structural loss, and parafunctional habits like bruxism. One of the critical prerequisites for PLV placement is the presence of sufficient sound enamel. When enamel is inadequate, the bond strength is significantly reduced, compromising the longevity and stability of the restoration [2,11,16]. 

Moreover, PLVs may not be suitable for cases involving extensive structural loss, such as large Class IV defects [2]. In such situations, the lack of a tooth-colored backing and the presence of unsupported porcelain increase the risk of fracture under functional stress [2,17].

Additionally, patients with parafunctional habits like bruxism or severe malocclusion may not be ideal candidates for PLVs, as excessive occlusal forces can increase the risk of veneer chipping or debonding. In such cases, occlusal analysis, protective night guards, or alternative materials such as high-strength monolithic ceramics should be considered [18].

An emerging alternative to full veneers is the use of sectional or partial porcelain veneers, particularly for localized corrections such as diastema closure. These restorations are gaining popularity due to their conservative preparation requirements. They preserve sound tooth structure by covering only the necessary portion of the tooth, thereby minimizing enamel reduction [19].

Wang and Zhao demonstrated that sectional feldspathic porcelain veneers can be successfully used in midline diastema closure with high esthetic integration and minimal tooth reduction [19]. This approach was not indicated in our case, as generalized fluorosis required comprehensive masking and esthetic enhancement across all maxillary incisors. In addition to influencing veneer selection, the fluorosis also posed specific challenges related to bonding and color matching [20]. While resin infiltration can improve mild fluorosis, moderate or severe cases typically require ceramic restorations to mask the discoloration and restore tooth morphology. Porcelain veneers, particularly lithium disilicate or feldspathic ceramics, have demonstrated superior outcomes in such contexts, offering both natural appearance and durability [20].

The treatment protocol included a diagnostic wax-up, mock-up, minimal enamel reduction of 0.5 mm, and final bonding. The result was a harmonious smile with ideal proportions and shade integration, confirmed at a one-week follow-up, during which the patient expressed full satisfaction with both the esthetic and functional outcomes.

This case reinforces the clinical efficiency of PLVs, especially in situations where orthodontic treatment is contraindicated or has failed, and highlights the growing role of PLVs as a minimally invasive alternative. While the short-term outcome was highly favorable, future clinical studies and long-term follow-ups are necessary to evaluate the durability and performance of PLVs in complex cases involving dental fluorosis or structurally compromised anterior teeth.

## Conclusions

The successful esthetic and functional management of MMD in a patient with very mild dental fluorosis highlights the clinical value of PLVs in complex restorative cases. By combining digital planning, diagnostic mock-ups, enamel-preserving preparation, and a meticulous adhesive protocol, the treatment achieved a conservative yet highly satisfying short-term result. This case demonstrates that PLVs offer an effective alternative when orthodontic correction is insufficient.

Beyond the closure of the diastema, this report underscores the importance of individualized treatment planning in cases involving enamel defects such as fluorosis, which present additional challenges in bonding and color matching. While the one-week follow-up confirmed the patient’s satisfaction and favorable functional integration, further long-term evaluation is needed to validate the predictability and durability of PLVs in such complex scenarios.
